# Comparative analysis of air permeability between UHPC and epoxy-coated normal concrete for hyperloop tube structures

**DOI:** 10.1016/j.heliyon.2024.e40598

**Published:** 2024-11-22

**Authors:** Dae Sang Kim, Ungjin Kim, Gebremicael Liyew, Chang-young Lee

**Affiliations:** a Korea Railroad Research Institute, 176 Cheoldobangmulgwan-ro, Uiwang-si, Gyeonggi-do 16105, Republic of Korea; b Department of Architectural Engineering, Chosun University, 309 Pilmun-daero, Dong-gu, Gwangju 61452, Republic of Korea

**Keywords:** Ultra-high performance concrete (UHPC), Epoxy-coated concrete, Air permeability, Hyperloop, Vacuum test

## Abstract

This study evaluates the air permeability of epoxy-coated normal concrete and ultra-high performance concrete (UHPC) for use in Hyperloop tube structures, where maintaining ultra-low air permeability is crucial to preserving the vacuum environment. While UHPC is recognized for its exceptionally low permeability due to its dense microstructure, this research explores epoxy-coated conventional concrete as a cost-effective alternative. Using a vacuum-based permeability test simulating Hyperloop's near-vacuum conditions, the study found that epoxy-coated concrete significantly reduced air permeability compared to uncoated concrete, with specimens coated on both sides approaching near-zero permeability. Although UHPC exhibited superior performance, the epoxy-coated concrete demonstrated air-tightness and was suitable for Hyperloop applications, offering a viable and economical alternative. These findings provide important insights for material selection, suggesting that epoxy-coated conventional concrete can effectively meet the stringent requirements for Hyperloop tube construction while balancing performance with cost-efficiency.

## 1 Introduction

The Hyperloop transportation system, which operates at high speeds in near-vacuum conditions, presents unique challenges in material selection for its tube structures. Among the critical challenges, maintaining ultra-low air permeability within the Hyperloop tubes is crucial to minimizing energy losses and ensuring the long-term stability of the vacuum environment [1,2]. The materials used for constructing these tubes must exhibit minimal air permeability while also being structurally robust, manufacturable at a large scale, durable, and resistant to environmental loading [3]. Additionally, given that the Hyperloop system may utilize superconducting magnets in combination with electrodynamic suspension and linear synchronous motors [4], it may be necessary to avoid conductive materials in tube structures. These specific requirements have driven research on ultra-high performance concrete (UHPC), as potential candidates for Hyperloop tubes due to their exceptional mechanical properties and low permeability [5,6].

UHPC is an advanced cementitious material known for its superior mechanical performance, such as compressive strength exceeding 150 MPa, ductility [7], impact resistance [8], and durability [9].A dense microstructure is produced by its low water to cement ratio and well-optimized particle packing, which highly reuduces pore size and improves durability, including low air permeability. Despite its low water content, UHPC maintains high flowability with the use of superplasticizers [10]. UHPC has been applied in various types of structures where durability and low permeability are critical, such as offshore [11,12] and railway infrastructures [13,14], and it has proven successful in bridge components [15] and innovative architectural applications [16]. Its low permeability of UHPC has been characterized under various conditions. For example, Dahal et al. reported that UHPC has significantly lower air permeability (10⁻^1^⁸ m^2^) under vacuum conditions compared to normal concrete and mortar (10⁻^1^⁶ m^2^), a result attributed to UHPC's densified microstructure and pore size reduction [17]. Furthermore, Tan et al. employed a nitrogen-based CEMBUREAU methodology to measure the gas permeability of UHPC and found that the incorporation of nanomaterials, including nano-SiO_2_ and nano-CaCO_3_, could lower the intrinsic gas permeability coefficient of UHPC to below 10⁻^22^ m^2^ [18]. This reduction is attributed to fine particles role in enhancing the density of the microstructure. These findings highlight UHPC's potential to meet the demanding requirements of Hyperloop tube construction. However, the high material cost and the inclusion of metal fibers in UHPC may limit its application in Hyperloop tube structures [19].

Although normal concrete has a lower material cost compared to UHPC, its larger and more interconnected pores result in higher air permeability [17]. The saturation degree and vacuum pressure have a significant role in the air permeability evaluation of the normal concrete sample [28]. Based on CEMBUREAU testing methodology, dry concrete samples exhibited a permeability coefficient between 10 and 25 x 10⁻^17^ m^2^ [29]. To address these challenges, some researchers have explored fundamental characteristics of conventional concretes and alternative materials to reduce their permeability. Katpady et al. confirmed the clear relationship between air permeability and compressive strength of conventional concrete, considering various water-binder ratio and curing condition [20]. According to Gardner et al., the gas permeability of both normal- and high-strength concrete significantly depends on the concrete grade rather than the curing temperature, even though different curing temperatures may lead to similar strength across different concrete grades [21]. Additionally, Güneyisi et al. demonstrated that the incorporation of supplementary materials, such as silica fume and metakaolin, into high-performance concrete can further reduce gas permeability, enhancing the material's resistance to environmental degradation [22]. However, there has been limited exploration of practical approaches, such as coating method to effectively reduce the air permeability of conventional concrete for Hyperloop tube applications.

To meet the specialized demands of Hyperloop tube structures, this study considers the application of epoxy-coating on conventional concrete. To evaluate the air permeability of concrete samples under extreme vacuum conditions, this study adopted the vacuum-based method proposed by Dahal et al. [17]. This method utilizes a two-chamber system where one chamber is subjected to vacuum conditions while the other is maintained at atmospheric pressure. Various coating methods and sample thicknesses were examined to assess their effects on air permeability.

The primary objective of this study was to experimentally investigate the impact of epoxy coatings on the air permeability of conventional concrete under the extreme vacuum conditions required for Hyperloop operations. While the air permeability of UHPC and epoxy-coated concrete has been studied under standard atmospheric conditions, there is limited research on their performance under the extreme vacuum conditions necessary for Hyperloop systems. The behavior of these materials in such environments is critical for ensuring the long-term viability of the Hyperloop. Moreover, there is a notable lack of comparative studies that directly assess the air permeability of UHPC against epoxy-coated normal concrete in a controlled vacuum environment, which is essential for determining the most suitable material for Hyperloop tube structures. This research seeks to address these gaps by conducting a comprehensive comparative analysis of the air permeability of UHPC and epoxy-coated normal concrete under vacuum conditions, providing valuable insights into their suitability for Hyperloop structures and offering a scientific basis for material selection that could lead to more efficient and durable systems.

## 2 Materials and experimental program

### 2.1 Materials and concrete mixes

Based on previous studies [23,^24^], the mix compositions for normal concrete (NC) and UHPC were determined as outlined in Table 1. For the NC specimens, Type I ordinary Portland cement (OPC) with a specific gravity of 3.12, river sand as fine aggregate, and coarse aggregate with a maximum size of 5 mm were used. The UHPC specimens were prepared with OPC, undensified silica fume that contains over 95 % SiO_2_, silica powder, two types of silica sand with average particle sizes of 105 μm and 370 μm, and a polycarboxylate-based superplasticizer in powder form. Additionally, 19.5 mm long, brass-coated steel fibers with a diameter of 0.2 mm were incorporated. The NC was mixed in accordance with ASTM C192 [25], utilizing a drum mixer. A laboratory planetary mixer was employed for the UHPC mixtures. After around 5 min of dry mixing the silica fume with the two types of silica sand, silica powder and cement were added, and the mixture was dry mixed for five more minutes. Then, water and superplasticizer were added gradually while the mixer was operating. Steel fibers were added to the mixture when it had attained the right consistency, and mixing was continued until the fibers were distributed uniformly.

**Table 1:** Mix proportions (kg/m^3^) of normal concrete (NC) and UHPC for the air permeability test.

Ingredients	NC	UHPC
OPC	400	857
Silica fume	–	214.3
Silica powder	–	214.3
Water	172.6	161.1
Superplasticizer	1.9	51.4
Steel fiber	–	78.5
Silica sand I	–	249.1
Silica sand II	–	581.3
River sand	791.5	–
Coarse aggregates	978.6	–

**Fig. 1:**
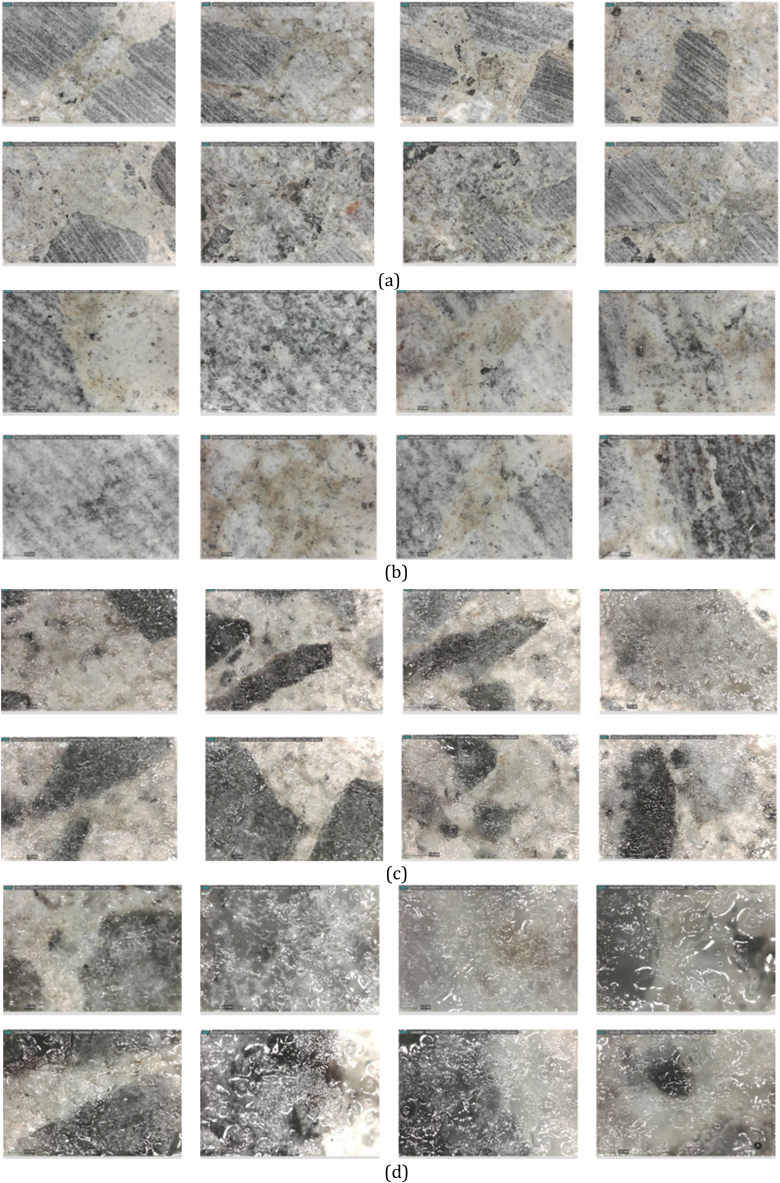
Concrete surface: (a) before epoxy coating (× 55 Resolution), (b) before epoxy coating (× 205 Resolution), (c) after epoxy coating (× 55 Resolution), (d) after epoxy coating (× 205 Resolution).

The fresh mixtures were poured into a 100 mm-diameter PVC molds and kept in a water tank for 24 h. The specimens were sliced to the required 15 and 30 mm thickness following a 7-day curing period. The specimens underwent an extra 21 days of curing, making it a total curing period of 28 days. Following the curing process, the specimens were dried in an oven set at 70 ± 5 °C for six days after the curing process, until the mass variation was less than 0.1 %.After drying, the specimens kept in a sealed condition until testing. The epoxy of DEVCON [S-209], which has 1500 psi or around 77500 torr strength capacity, was used with a hardner and resin proportion of 1:1 by wt%. The epoxy was evenly applied to the designated surface for the air permeability test to evaluate its effect on the impermeability of samples. The epoxy coated samples were kept in the oven for 24 h at 60° temperature to increase the hardening process. Fig. 1(a–d) shows the concrete surfaces before and after the epoxy coating.

### 2.2 Experimental procedure

**Fig. 2:**
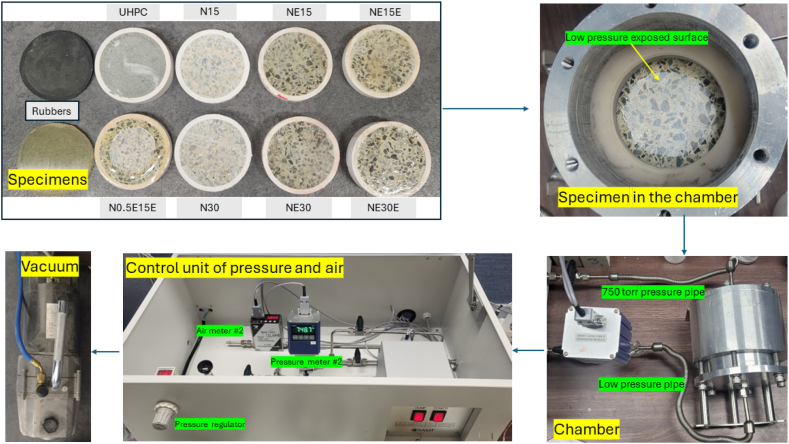
Experimental setup for air permeability evaluation.

**Fig. 3:**
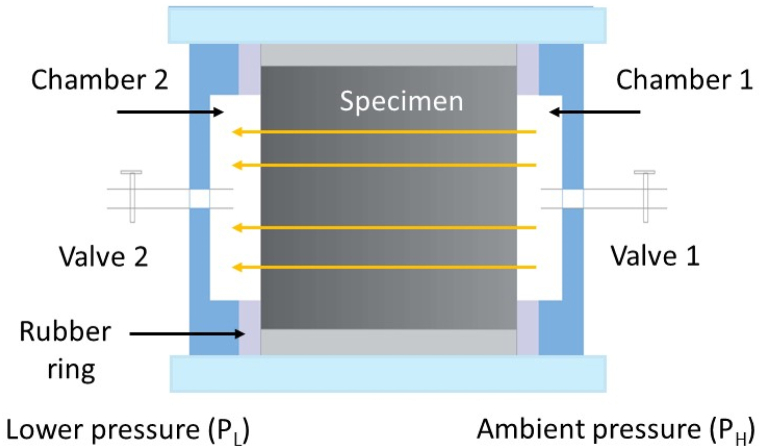
Illustration for air permeability in the chamber including specimens.

The experiment was conducted by applying a vacuum pressure on the pipe that connects the control unit and sample holder pressure. The sample was subjected to lower pressure on one side of the sample (P_L_) and atmospheric pressure on the opposite side (P_H_), 750 torr. During the experiment, the range of lower pressures for *P*_*L*_ was depend on the sample type. Mortar samples can drop the pressure around 15 torr. However, the concrete and UHPC samples can lower the pressure between the range of 12–13 torr. Although the original capacity of the machine was able to achieve 0.001 atm (0.76 torr), the experiment encountered leakage issues within the apparatus. As a result, achieving the target pressure took an extended period of over 3 h. Therefore, 12–13 torr was selected as the starting pressure for analysis. Fig. 2 shows the experimental setup and procedure of the air permeability evaluation. Fig. 3 illustrates the direction of airflow during the air permeability test.

### 2.3 Variables investigated

**Fig. 4:**
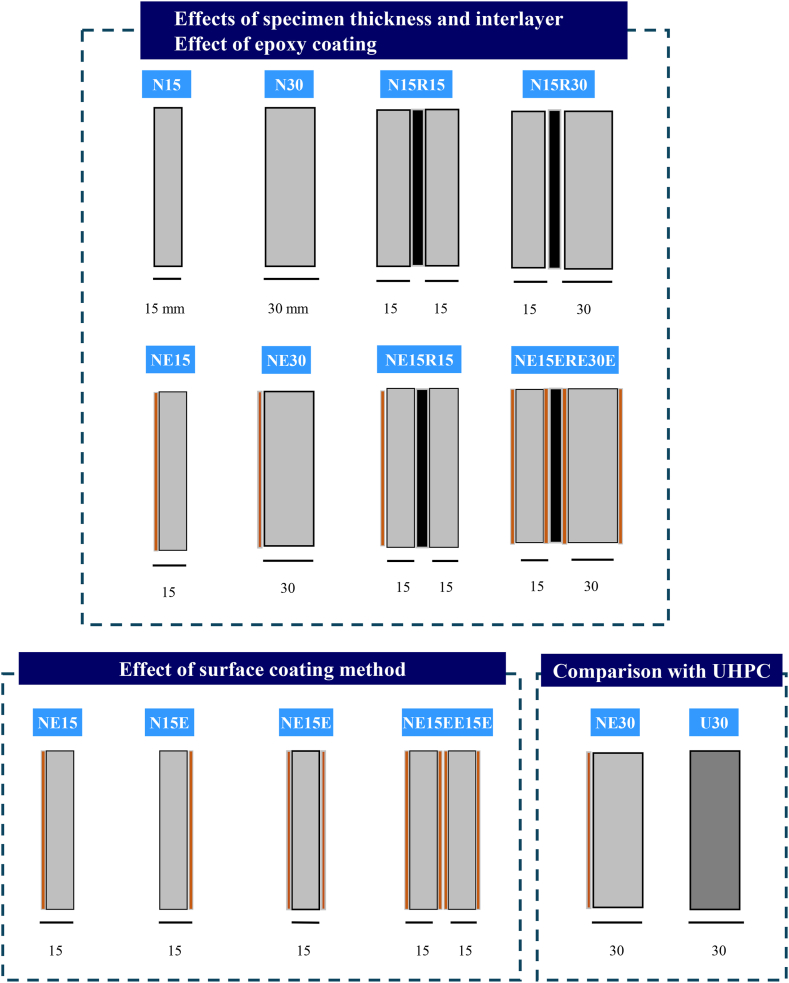
Details of the experimental program (Note. E: Epoxy coating, R: Rubber layer, N: normal concrete, U: UHPC).

As shown in Fig. 4, this research focuses on four key variables: specimen thickness, interlayer, coating surface, and concrete grade. Each series is designated by specimen thickness (15 mm or 30 mm), interlayer (R for rubber interlayer), coating surface (E for epoxy coating), and concrete grade (N for normal concrete and U for UHPC). Additionally, the orientation of the coating surface is considered; for example, in the NE15 series, the coating surface faces the vacuum pressure.

### 2.4 Experimental setup and gas permeability coefficient

**Fig. 5:**
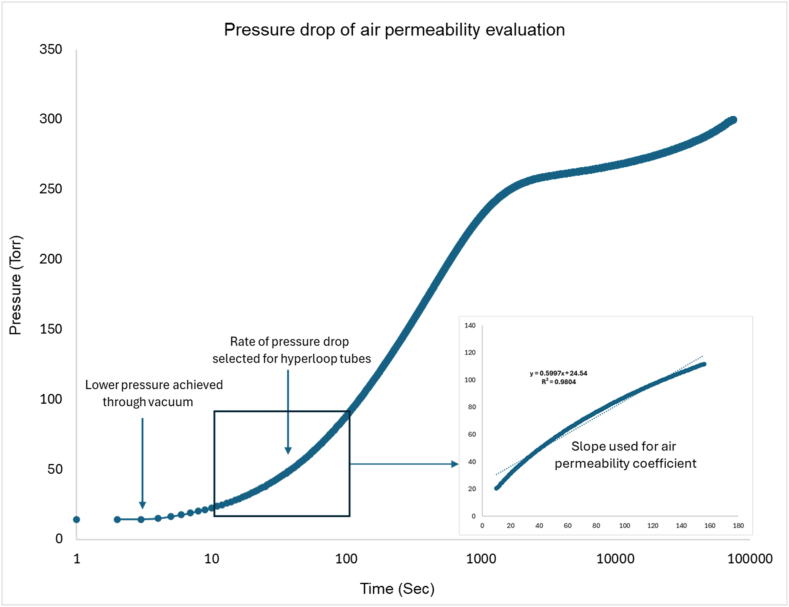
Pressure drop and slope selection for the analysis of air permeability coefficient.

A vacuum-based permeability apparatus [23] was employed to assess air permeability of specimens under vacuum conditions. The testing setup is designed to endure extreme vacuum pressures and utilizes a two-chamber system with the lower chamber connected to a vacuum pump. The pressure in the lower chamber was stabilized between 10 and 15 torr, while the upper chamber remained at atmospheric pressure. Air permeability was determined by tracking the pressure increase in the lower chamber over time. Fig. 5 shows a typical example of a pressure drop while monitoring the pressure changes.

The gas permeability coefficient (*K*_*a*_) is a critical parameter for evaluating the impermeability of materials, particularly in applications such as Hyperloop tube structures, where maintaining a near-vacuum environment is essential. The gas permeability coefficient was determined using the formula proposed by Hamami et al. [26], which has proven effective in experiments conducted under near-vacuum conditions [23].

The permeability test was conducted using a vacuum-based apparatus, which can able to withstanding extreme vacuum pressures. The test setup utilized a two-chamber system, where the lower chamber was connected to a vacuum pump. While the upper chamber (PH) stayed at atmospheric pressure (750 torr), the lower pressure cahmber (PL) was bale to stabilize between 10 and 15 torr.

The calculation of the gas permeability constant *K*_*a*_ was based on the change in pressure over time in the lower chamber, as derived from Darcy's law. Fig. 5 shows a typical time range used for calculating the gas permeability constant. The following equation (1) was used to determine the apparent gas permeability (*K*_*a*_):

(1) $$K_{a}=\frac{2nL}{\left(P_{H}^{2}-P_{L}^{2}\right)A}V_{L}\frac{dP_{L}}{dt}$$

Where, *K*_*a*_ is the apparent gas permeability (m^2^), *n* is the air dynamic viscosity (Pa·s), *L* is the thickness of sample, *P*_*H*_ is the pressure in upper chamber (750 torr), *P*_*L*_ is the pressure in lower chamber (15 torr), *V*_*L*_ is the volume of chamber 2 (m³), *A* is the sample cross-sectional area (m^2^), *dP*_*L*_/*dt* is the rate of pressure change in the lower chamber (torr/s).

This equation derives from the principle that gas will always flow from a region of high pressure to a low pressure through a permeable medium. The flow rate and permeability depend on the medium's characteristics, as described by Darcy's law.

The formula used integrates the thickness of the sample and accounts for gas flow changes in the second chamber can be determined using equation (2), as governed by the change in mass flux (*Q*_*m*_) over time:

(2) $$Q_{m}=\frac{K_{A}}{\eta }\frac{M}{RT}P\frac{\partial P}{\partial x}A$$

where, *Q*_*m*_ is the mass flux (kg/s), *P* is the gas pressure (Pa), ∂x is differentiated by the sample thickness (m), *T* is the gas temperature (K), *M* is the gas molar mass (kg/mol), R is the ideal gas constant (J/mol·K).

Combining the gas flow and mass flux equations presented on equations (1), (2)), leads to the final form of the permeability equation. This method provides a reliable approach to measuring the gas permeability of materials under extreme vacuum conditions. The use of Hamami's formula allows for accurate permeability coefficient calculation of the of the gas *K*_*a*_ in near-vacuum conditions. The experimental approach effectively measures how materials such as UHPC and epoxy-coated NC perform under such conditions, offering valuable insights for optimizing material selection in applications like Hyperloop tubes. It should be noted that due to the inherent limitations of the test device, minor air pressure leakage occurs during the test. To address this issue, a 5 cm thick PVC specimen was used to analyze the inherent leakage of the test device. The same value (0.706 × 10^−18^ m^2^) was obtained from three repeated tests, and in the actual analysis, the gas permeability constant was calculated by subtracting this value from the results of all concrete specimens. In most cases, the experiments were conducted multiple times using the same specimens, and the averaged results were used for the analysis.

## 3 Results and discussion

**Fig. 6:**
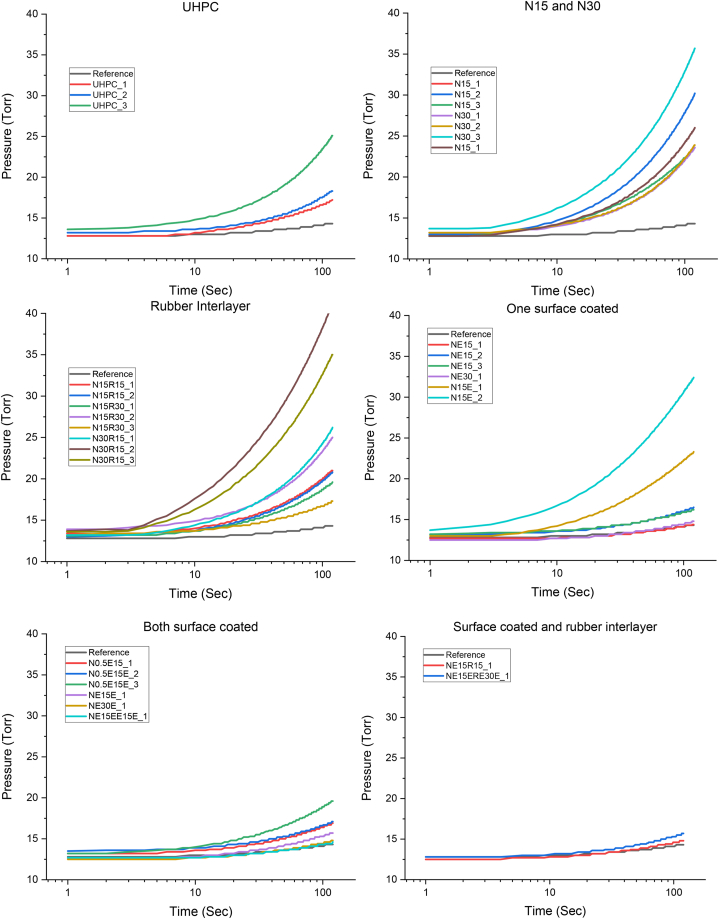
Pressure changes of non-coated normal concrete, coated concrete, and UHPC samples over time.

**Fig. 7:**
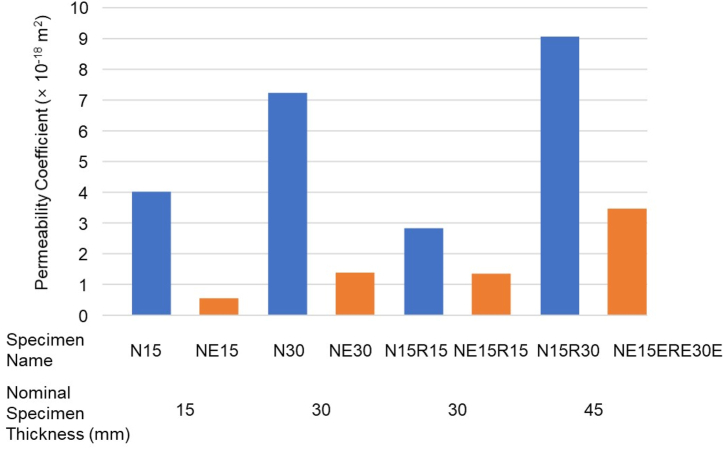
Effect of specimen thickness and epoxy coating on gas permeability coefficient.

Representative examples of pressure changes over time, as shown in Fig. 6, demonstrate that the testing setup reliably measures pressure variations under vacuum conditions, with minimal discrepancies observed across the specimens. The gas permeability results, along with two critical parameters—specimen thickness and epoxy coating efficiency—are presented in Fig. 7. The effect of specimen thickness is evident when comparing four sets of specimens: N15 and N30, N15R15 and N15R30, NE15 and NE30, and NE15R15 and NE15R30. In addition to thickness, the specimen surface condition plays a significant role especially for non-epoxy coated samples. Although the materials used—such as concrete, coating, and rubber—are essentially the same, the gas permeability coefficients showed a clear trend of increasing as specimen thickness increased. This trend may be attributed to the inherent characteristics of the test setup, where the probability of leakage rises with specimen thickness. Consequently, direct comparisons between specimens of different thicknesses may lead to unrealistic conclusions.

Furthermore, the rubber interlayer between the concrete samples and the epoxy coating significantly affects the permeability coefficient. For samples with a nominal thickness of 30 mm, the insertion of an interlayer in the middle of the sample resulted in an average reduction of 31.6 %. It is interesting to note that the effect of the interlayer is more pronounced in samples without epoxy coating, as the coated samples already exhibit minimal permeability coefficients, highlighting the effectiveness of the coating materials and method. When directly comparing specimens with and without epoxy coating, the reduction in permeability is even more apparent, with an average drop of 70.1 %. Therefore, it can be concluded from Fig. 7 that epoxy coating is a highly effective method for improving the gas permeability coefficient, and further enhancement can be achieved by incorporating an interlayer.

**Fig. 8:**
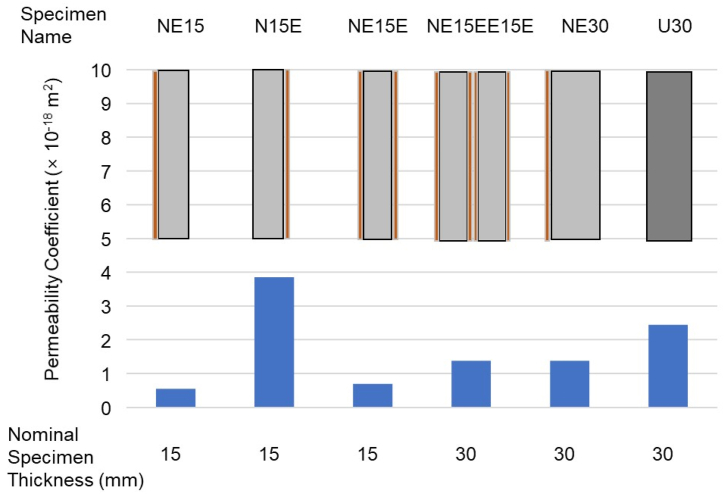
Effect of surface coating method on gas permeability coefficient and comparison with UHPC.

The effects of coating direction and coating methods are illustrated in Fig. 8. As shown in Fig. 3, the two surfaces of the concrete are exposed to critically different pressures—ambient pressure and pseudo-vacuum pressure. Therefore, even though the same materials are used for both the main structure and the coating layer, the coating efficiency is not identical. The comparative results between the NE15 and N15E samples clearly indicate that applying the coating towards the vacuum pressure, i.e., on the inner layer of the tube structure, is significantly more effective, resulting in an 85.5 % decrease in permeability coefficient. When comparing one-sided coating to multiple-layer coatings, including two-sided coating, the results suggest that multiple layers do not offer significant benefits over a single coating applied towards the vacuum direction. This can be attributed to the fact that a single layer of epoxy coating is robust enough to effectively block gas flow under vacuum conditions, provided it is properly adhered to the main structure.

The effectiveness of the coating method is further evaluated by comparing it to the most advanced cementitious material, UHPC. As highlighted in the literature [17,27], due to its extremely dense microstructure UHPC exhibits superior gas impermeability compared to other cementitious materials. A direct comparison between NE30 and U30 samples shows that normal concrete with a single-layered epoxy coating achieved a permeability coefficient comparable to that of UHPC. Therefore, it can be concluded that a single-layered epoxy coating applied towards the vacuum pressure is a practical and efficient method for reducing gas permeability.

## 4 Conclusions

This study comprehensively evaluated the air permeability of UHPC and epoxy-coated normal concrete for potential application in Hyperloop tube structures, where maintaining ultra-low air permeability is essential for sustaining a vacuum environment. The results demonstrate that while UHPC, with its dense microstructure, exhibits superior air impermeability, epoxy-coated normal concrete also presents a highly effective and more cost-efficient alternative.

The experiments revealed that applying a single-layer epoxy coating reduced the gas permeability coefficient of normal concrete by an average of 70.1 %, with an additional 85.5 % reduction when the coating was applied towards the vacuum pressure. Furthermore, the incorporating of a rubber interlayer decreased gas permeability by 31.6 %, particularly for uncoated specimens. The findings also indicate that epoxy coatings, especially when applied on both sides of the concrete and oriented towards vacuum pressure (1.39 × 10^−18^ m^2^), significantly reduce air permeability, bringing it close to the levels observed in UHPC (2.46 × 10^−18^ m^2^). The introduction of epoxy coatings resulted in a substantial reduction in permeability, with coated samples achieving near-zero permeability under extreme vacuum conditions. A single-layer epoxy coating, when properly adhered to the concrete surface facing vacuum pressure, was found to be sufficient in achieving the desired air impermeability, eliminating the need for multiple layers, which offered no significant additional benefits.

In conclusion, while UHPC remains the gold standard for low-permeability applications, epoxy-coated normal concrete emerges as a viable alternative for Hyperloop structures, offering a combination of excellent performance and cost-effectiveness. These insights are crucial for material selection in Hyperloop tube construction, guiding future research and development toward more efficient and sustainable solutions. Future studies should further explore these findings by investigating the long-term durability of the epoxy coating under actual vacuum conditions and repeated passage of subsonic capsule trains. Additionally, potential issues such as internal cracking between the concrete and coating layer, or within the coating layer itself due to structural displacements of the tube during operation, must undergo thorough validation to ensure the system's safety.

## CRediT authorship contribution statement

**Dae Sang Kim:** Writing – original draft, Visualization, Validation, Funding acquisition, Formal analysis, Conceptualization. **Ungjin Kim:** Writing – original draft, Supervision, Project administration, Methodology, Conceptualization. **Gebremicael Liyew:** Writing – review & editing, Validation, Software, Formal analysis, Data curation. **Chang-young Lee:** Writing – review & editing, Validation, Supervision, Resources, Conceptualization.

## Data availability

Data will be made available on request.

## Declaration of competing interest

The authors declare that they have no known competing financial interests or personal relationships that could have appeared to influence the work reported in this paper.
